# Piloting a framework to explore the impacts of public health workforce capacity-building initiatives

**DOI:** 10.3389/fpubh.2025.1677187

**Published:** 2025-12-03

**Authors:** Cheyanna Frost, Jeanne W. Lawless, Donna Leong, Genevive R. Meredith

**Affiliations:** 1 Cornell University Public Health Program, Ithaca, NY, United States; 2 Cornell University Health Impacts Core, Ithaca, NY, United States; 3 Department of Public & Ecosystem Health, Cornell University, Ithaca, NY, United States

**Keywords:** public health, public health workforce, evaluation, workforce development, outcomes evaluation

## Abstract

**Introduction:**

To reinforce and re-build the public health workforce, many capacity building interventions are in place. While pre-post assessments are often used to describe short-term outcomes, methods to assess and describe longer-term outcomes and impacts are wanting. Our work aimed to help close this gap by exploring ways to assess and describe longer-term outcomes, including how capacity gains contribute to new actions taken by individual workers and organizations in support of public health goals. We hoped this work might inform development of an evaluation framework able to measure outcomes and impacts of public health workforce capacity-building initiatives.

**Methods:**

Building from short-term outcomes data demonstrating changes in participant capacity (knowledge, skill, confidence), we used a multiple case study design to explore outcomes resulting from the use of the online *Public Health Essentials* (PHE) capacity building intervention. We conducted in-depth interviews with a purposive sample of eight PHE graduates (Summer 2023-Spring 2024) to elucidate both medium-term outcomes and potential longer-term impacts. Qualitative interviews were coded and analyzed using *a priori* and emergent themes (Spring-Fall 2024).

**Results:**

Interview analysis revealed outcomes grouped into 13 themes. PHE graduates described how capacity growth influenced seven individual capabilities and their ability to take collective or shared actions in three areas. Further, they described their ability to influence changes in conditions in three areas critical public health: health equity, social determinants of health, and prevention.

**Conclusion:**

Evaluating longer-term outcomes and impacts of capacity building interventions is crucial to both improve and justify public health workforce development initiatives, particularly as prevention and population health needs persist. We posit that evaluations will be more effective if standardized methods are used across interventions, and if there is a greater push to share and publish results. We present a conceptual framework that may inform and guide future evaluation and process improvement efforts.

## Introduction

The health and wellbeing of people in their communities and ecosystems is *Public Health* (1–3). This state is assured by the collective work of people and organizations influencing the policies, practices, and systems that work to prevent disease or injury, promote health, and prolong life (1–4). To achieve this, an interdisciplinary workforce is required, equipped with right-fit knowledge, skills, and capacities to support delivery of core functions and essential services (1, 2, 4–6).

Prompted by the COVID-19 pandemic, and in anticipation of future public health needs, governments, allied organizations, and working groups around the world have developed competency frameworks and guidance to support the development and growth of the public health workforce (1, 2, 7, 8). Together, these have informed and spurred refinements to public health education frameworks (e.g., bachelors, masters, doctoral degrees in public health) (9–11), and have expanded capacity-building interventions and new methods for public health worker recruitment, in-service training, and retention (10, 12–14). For example, in the US, public health workforce assessment and enumeration efforts have shown that the public health system is understaffed (4), and that the current public health workforce has skills gaps and training needs (15). During and following the COVID-19 emergency, many initiatives were developed to help backfill and meet critical public health workforce needs through hiring, training, and up-skilling (16, 17). The outcomes of these efforts, however, are not yet well elucidated (15).

### Building public health workforce capacities

Many approaches are used to build capacities among public health workers, such as education in degree-granting programs, in-service training, online training, and mentoring for new and existing workers (1, 18–21). The World Health Organization (WHO) reports that capacity development is a result of learning content delivered, organization of the content, teaching methods used, learning and learner experiences, and the methods of assessment used with the learners (1). To support this, WHO working groups have defined essential public health functions, subfunctions, and services; provided guidance to help strengthen competency-based training and education oriented toward the delivery of the essential public health functions; and mapping and measuring the diversity of occupations involved in delivering—and needed to ensure delivery of—these functions (1, 2, 7).

Methods and models used for public health workforce assessment and development in the US align with the WHO guidance. For example, the Public Health Workforce Interest and Needs Survey (PH WINS) reliably assesses workforce training needs and skill gaps among the governmental public health workforce (15, 22), helping to inform education and capacity-building efforts (6, 17, 23–27). Workforce development strategies in the US largely focus on building knowledge and skills in “specialized skill” domains (e.g., epidemiology), as well as the *Core Competencies for Public Health Professionals* (8), and the *Public Health Strategic Skills* (28), in both degree-granting public health education programs (9–11), and through workforce development/ training efforts. These frameworks aim to build foundational, cross-cutting capacities in areas such as Effective Communication; Data-Based Decision Making; Cross-Sectoral Partnerships; Leadership; Systems Thinking; Community Engagement; Change Management; and Program Planning, equipping public health workers and organizations to ensure *The 10 Essential Public Health Services* in their communities (29). Additional influences on public health capacity building in the US focus on ensuring public health programs are aligned to community needs, re-building public trust in public health, and collaborative practice for collective impact (3, 6, 17, 30, 31).

### Translating capacities into action

While numerous approaches are being used to build capacities among the public health workforce in the US and globally, there is a paucity of reports on if or how workforce training needs are being met, and if or how increased workforce capacity manifests in real-world settings (32). We posit that a focus on this is critical. At present, despite a concerted focus on workforce development, literature to help inform evaluation of outcomes is scant. For example, repeat rounds of PH WINS show no significant change closure of skills gaps despite the broad availability of capacity building resources (15). Further, while there is some literature reporting on the short-term effectiveness of trainings (e.g., knowledge or skill-gains pre-post training intervention) (14, 33, 34), a 2023 study on COVID-19 contact tracing scale-up stated that their work was the “first comprehensive analysis” that aimed to illustrate the interrelationships between the capacities, capabilities, outcomes, and impacts (35). This may be because impacts are not easy to identify—especially in the short-term—and are highly context-specific. Evaluation of longer-term outcomes or impacts appear more prevalent in health care fields, where the application of expanded capacities can be observed by a supervisor or an evaluator (36–38), but even an implementation study exploring workers’ expanded capacity to address HIV and TB identified public health impacts as an evaluation criterion that was hard to measure. The research team reported several challenges in monitoring and reporting on public health impacts, including building systems for monitoring impacts, securing resources, and engaging leadership (39).

We propose that there is an opportunity for expanded investigation around public health workforce development, globally. More robust, shorter-term evaluations of workforce development efforts are needed to measure and improve intervention effectiveness. Additionally, we posit that evaluation of capacity building interventions must go a step further and explore if or how gained capacity translates into *mid-and-longer-term outcomes* and *collective public health benefits or impacts.* And, while this may need to be context specific, we posit that there may be standard framing or frameworks that could be used. To test the feasibility of this, we designed an initial exploratory process focused on one cohort of learners.

### Filling the gap—public health essentials as a case study

As a part of the multi-pronged approach to reinforce the public health workforce in the US, our team developed and deployed a capacity-building intervention. The Public Health Essentials (PHE) curriculum was designed to build and assess competence in 54 skill areas, and developed using pedagogical and behavior change theories (14). PHE is a cohort-based, facilitated, on-line intervention that builds competence and confidence among new and existing public health workers over a 15- to 20-week period (14). The approach aims to rapidly equip people working in public health roles with the knowledge and applied skills needed for foundational public health work (14). PHE comprises 75 h of content, and all learning and assessment is completed in an asynchronous classroom. Learners access and complete lessons and graded competency assignments and receive developmental feedback from expert facilitators (14).

Prior PHE-focused research focused on understanding outputs (e.g., program uptake, completion rate) and short-term outcomes (e.g., changes in worker competence and confidence); this work showed that PHE graduates demonstrate significant competency gains, and report an ability to apply knowledge and skills acquired to their work (14, 40). However, these studies did not get at the *So What*: does expanded capacity translate into actions to advance public health, and if so, how. This is what we set out to explore in this project.

**Figure 1:**
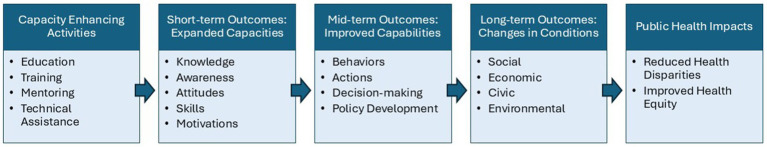
*A priori* theory of change—translating capacity building into action.

To explore this “so what” of a capacity building intervention that shows short-term effectiveness, we used a logic model/pathway to impacts approach to expand our *a priori* theory of change. We knew that activities [the PHE intervention] had led to short-term outcomes [improved knowledge, skill, motivation], but we had only hypothesized that those might lead to mid-term outcomes [changes in capabilities manifest as behaviors, actions] and longer-term outcomes [changes in conditions] that could result in community health impacts (Figure 1). As PHE was designed to build *Core Competencies* and *Public Health Strategic Skills*, we further hypothesized that these capacities could equip a worker with stronger capabilities aligned with the essential services of public health, allowing them to contribute to changing systems and processes to improve wellbeing.

## Methods

We used a qualitative case study approach to assess this: how capacity gains among PHE graduates are translating into public health-focused outcomes (Cornell IRB Protocol #00147810). PHE graduates who had demonstrated increased competence (expanded capacities) across 54 skill areas post-PHE were qualified to participate. To control for possible bias inherent from working within a public health department, we applied purposive sampling to a national cohort of community-based public health workers not working in governmental public health (*n* = 58). Eight respondents were selected based on geography (a variety of US states), work location (community based), and responses to a screening survey about how they were applying PHE-supported learning into their current work (articulate a community public health need that they were focused on). Interviewees provided both written informed and verbal consent.

The interview protocol was developed and piloted by members of our research. Interviews were conducted Summer 2023-Spring 2024 by (DL, JL, CF) and focused on five key questions related to (a) the focus of their public health work, (b) their motivation to do this work, (c) what actions they take to achieve this, (d) what outputs and outcomes they are seeing, and (e) how PHE-derived skills have helped them do this work.

Interviews were recorded (Zoom, Version 5.15.7 or 5.17.2); transcripts were generated (Rev Transcription Services), cleaned (JL), and coded for themes (Dedoose version 9.2.006) by two qualitative researchers (JL, CF) using a shared codebook that was developed in an iterative manner. Initial codes focused on actions or behaviors, and were proposed *a priori* from our literature review (including *Core Competencies, Strategic Skills, Essential Services*); these were expanded and clarified during pilot interviews using a modified hybrid approach (41) where two coders (JL, CF) assessed alignment and divergence of code application, discussed code application strategies and definitions, and developed a code book. Consultation with other team members regarding any discrepancy helped solidify consistent application of codes. Between-coder triangulation was used until consensus in code application was reached.

Qualitative analyses of the coded transcripts were conducted Spring-Fall 2024 (JL, CF). Code application frequencies were used to explore cross-case themes (Dedoose, Version 9.2.006) and to identify emergent capabilities being applied to modify conditions to improve community health.

## Results

### Expanded capacities

All respondents (*n* = 8) were women and all reported working in the field of public health for at least 3 years. All respondents worked in rural counties in the U. S., including in NY (*n* = 3), NE (*n* = 2), WI (*n* = 1), AR (*n* = 1) and NC (*n* = 1). Each respondent recalled having completed PHE in the preceding 1–2 years and having appreciated knowledge and skill gains (*expanded capacities*) as a result. Respondents reported participating in PHE for a variety of reasons, including broadening their understanding of public health and learning new frameworks to apply to their public health work.

*A priori*, we hypothesized that the PHE graduates might be able to describe how their PHE-supported changes in knowledge and skill (short-term outcomes) have led to changes in their capabilities (mid-term outcomes), helping them work toward community impacts. Via the coding process, two distinct types of “improved capabilities” were noted: behaviors or actions taken by an individual (*Individual Capabilities*), and behaviors or actions taken by an organization (*Collective Actions*); respondents also described some outcomes (*Changes in Conditions*) they hoped to see as a result of their actions.

### Improved capabilities

When asked to describe how the knowledge and skills gained in PHE influenced their work, 224 excerpts were coded as *Individual Capabilities*. In general, individual capability codes focused on the behaviors or actions an individual takes in their day-to-day public health work. Based on our *a priori* framework, we envisioned hearing about individual actions aligned with the *10 Essential Services*, but codes ended up focusing on a mix of *Core Competencies*, *Strategic Skills*, and *Essential Services*.

Across the eight cases, seven *Individual Capabilities* themes were noted (Table 1), including investments in External Collaborations (*n* = 56), Communication (*n* = 40), Program Planning (*n* = 37), and Leadership (*n* = 30). All themes except Advocacy were noted by at least 75% of the cases.

**Table 1:** Frequency of themes coded in interview transcripts.

	Individual capabilities	Frequency of codes	Number of cases
Mid-term outcomes	External collaborations	56	8
	Communication	40	7
	Program planning	37	8
	Leadership	30	8
	Systems thinking	25	7
	Assessment & data analytics	19	6
	Internal collaborations	17	7
	Collective actions	Frequency of codes	Number of cases
	Strengthen public health systems	94	8
	Align programs to community needs	41	7
	Improve confidence/trust	7	3
	Changes in conditions	Frequency of codes	Number of cases
Long-term outcomes	Improved health equity	39	7
	Improved social determinants	29	7
	Improved prevention	14	8

*Collaboration* was coded when respondents reported translating PHE-skills building to invest in sustained, long-term, ongoing reciprocal partnerships where teamwork increased or workloads were shared. This was further segmented into collaborations with others within their workplace (*Internal Collaboration*), vs. collaboration with community members or organizations (*External Collaboration*). *Program Planning* was coded when respondents reported translating skills to create, champion, and/or implement policies or programs to address health needs. See sample quotes in Table 2.

**Table 2:** Exemplar quotes from interviewees highlighting individual capabilities, collective actions, and changes in conditions.

Theme	Exemplar Quote
Individual capabilities	
Collaborations	*“I really think that it’s everybody, all the different nonprofit partners working together, looking at that data and talking about the specific needs and how they could address ‘em… that is what really helps the most.”* *“There are so many organizations working on this and so many nonprofits and so many grants and we are almost tripping over each other. We are tripping over each other. And so what a lot of this work came out is how do we collaborate, how do we work together? How do we get the right people in the room to be doing this work?”*
Program planning	*“…in terms of public health, that was an area that I did not have a whole lot of education behind. [PHE helped] fill in those gaps…to help with program planning for the future.”* *“One thing I realized was that pumping out nutrition information really is not helpful…. I really need to be looking at where these people are at, and especially culturally, because a lot of the recipes and the resources I have aren’t culturally sensitive.”*
Communication	*“Yeah, [it helped me] just take a step back and not come in with my preconceived notions about why something’s occurring, but just to be more inquisitive.”* *“[I now consider] how do I frame it so that they can see the benefit…it’s then having those conversations with those who maybe do not see the benefit… being able to communicate effectively.”*
Systems thinking	“*I do think that that is the shift… I feel like I experienced quite a bit now…[it] is like I’m constantly trying to better paint the picture of clientele in terms of their needs. Again, where are they living? Do they have access to these things? What’s their financial situation? All these little things that play a part [in] health.”* *“… all of those barriers that exist just within the environment that we have to address first before we can get to that level of nutrition education… there’s so many other things that we need to address. And I started thinking … there’s so much more that I can do with this, or there’s just a different way that I can approach some of these conversations rather than thinking about it so linearly.”*
Leadership	“*I feel like taking PHE gave me confidence… and prepared me to be in a position to be invited to the table*” and *“I have [the] LARA [tool] just sitting on my desk […] I just remember it being so valuable that I wanted to have it in an easily accessible place so I could refresh my memory about it all the time.”* “*We’re using those tools in conversations…really helpful to me just navigating difficult conversations.”*
Assessment and data analytics	*“I really appreciated that [PHE] forced a bunch of people from our area to talk about all the needs in our area together and to talk about the information that we have on our area”* *“We [now’ have an evaluation specialist who helped with creating that survey so that we can get at what we are really wanting to know about participation].”*
Collective actions	
Strengthen public health systems	“*[now] there are other counties that are doing this too…. using our model.”* “…*we did a fruit and vegetable prescription program class where [seniors in a residence] were prescribed to take a class and then they would get vouchers for fresh produce…and then go straight to the veggie van after the class. It worked out really well and the farmers got more money and more people using their service].”*
Align programs to community needs	*“The whole purpose of it is the young people, it’s their voices, it’s their analysis of their community needs and their ideas that we as the adults act as guides to guide them through the process of thinking about all those things and making decisions and coming up with an action plan.”* “*I guess for me, I hope that I continue to keep my eyes open to the needs of a specific group and not just generalize my information, but really kind of thinking through how I can individualize based on the needs of the audience I’m presenting to.”*
Improve confidence/trust	*“Long-term, I would hope that it is helping our community participants be more trusting, more willing to participate in the programs or getting health services or whatever the case may be, preventative care with breast cancer exams, etc.”* *“I hope that it is helping our community participants be more trusting, more willing to participate in the programs or getting health services or whatever the case may be, preventative care with breast cancer exams, etc.”*
Changes in conditions	
Improved health equity	*“We started talking about how to be more equitable within what we offer to our constituents, but being able to bring that example to the larger community and saying, these are some of the ways that we are trying to, what are some other ways that all of our can be doing some of these practices in your work.”* *“We hope to…help combat the disparities that we see in these communities. [We want to] be able to link them to the resources that they need to get the support [and] the healthcare services that they need.”*
Improved social determinants	*“Anybody can participate with the veggie van. But… we try to pick sites that do not have a lot of farmers that accept Farmer’s Market Nutrition program checks, and we do target rural locations where it’s hard to get to fresh produce and we target senior living or people in Section eight housing facilities as well.”* *“I happen to have a passion around the need for childcare and meeting that need based on my own experiences. Likewise, I’m on a transportation work group that’s exploring rural transportation resources and how can we build up the resources that are available here for our local residents.”*
Improved prevention	*“There are a lot of different strategies… [we are hoping] to help people be healthier and more preventive versus reactive.” ….”So that’s the broad scope: how do we help prevent chronic diseases?”* *“But a lot needs to be done in the schools because children, when they learn early, hopefully they keep those habits.”… “I think a lot of it really flowed into targeting areas where children are affected, specifically because of the dental health issue. If we can catch work on dental health early on, then they are more likely to have a healthier life.”*

*Communication* was coded when respondents reported translating PHE-skills building to develop or disseminate information to help inform and educate, including working with community stakeholders to ensure culturally and linguistically appropriate communications. *Systems Thinking* was coded when respondents reported translating skills to engage cross-sectoral partners to understand and explore inter-related systems, including when working to develop a shared vision of how to better collaborate to address public health needs.

*Leadership* was coded when respondents reported translating PHE-skills building to lead or support teamwork, collaboration, or action. *Assessment and Data Analytics* was coded when respondents described translating skills to collect data to understand needs or opportunities, or to use data to guide actions.

### Collective actions

When asked to report on how the knowledge and skills gained in PHE influenced their work, 152 excerpts were coded as *Collective Actions*. In general, collective action codes focused on the reason a person, an organization, or a group took or is taking action; these appeared to focus loosely on the *Essential Services*. Across the eight cases, three *Collective Action* themes were noted (Table 1), including Strengthen Public Health Systems (*n* = 94), Align Programs to Community Needs (*n* = 41), and Improve Confidence/Trust (*n* = 17). All themes except Improve Confidence/Trust were noted by at least 75% of the cases. *Strengthen Public Health Systems* was coded when respondents reported translating PHE-skills building to broaden the networks of organizations contributing to public health, working to enhance capacity among those working to support public health, and improving cross-sectoral collaborations to meet a need. *Align Programs to Community Needs* was coded when respondents reported translating skills to help adapt or improve programs—based on qualitative or quantitative data—to better meet the needs of a community. *Public Confidence/Trust* was coded when respondents reported translating skills to aim to [re]build trust in public health and the public health system. See exemplar quotes in Table 2.

### Changes in conditions

When asked to describe why they are focused on the projects highlighted in the interviews, all respondents reported a focus on at least one “*Changes in Conditions*” related to influencing the health and wellbeing of the communities they serve. Each respondent was able to clearly articulate a community public health need that their work focused on (e.g., improving food access, food security, fruit and vegetable consumption, health-supporting behaviors, youth development, and community development). Some 82 excerpts were coded across the eight cases, three *Changes in Conditions* themes were noted (Table 1), including Improved Health Equity (*n* = 39), Improved Social Determinants (*n* = 29), and Improved Prevention (*n* = 14). *Improved Health Equity* was coded when respondents reported taking actions to ensure inclusion or improved diversity, or to specifically improve health equity. *Improved Social Determinants* was coded when respondents reported explicitly addressing needs related to a determinant of health, such as housing, transportation, quality food access, education, or quality health care. *Improve Prevention* was coded when respondents reported explicitly working to prevent disease or harm or disability. Several respondents talked about working with youth to develop healthy lifelong habits, such as eating healthy foods and going to the dentist.

Across the interviews, respondents also spoke to the “why” behind their work, or what motivates them invest to in public service. Respondents focused on altruistic themes: *“I want to implement programs that will make a difference.”* “*I would love just to see people eating healthier and having better access to this produce.”*

### Cross theme analysis

PHE was designed to build *Core Competencies* and *Public Health Strategic Skills* and we hypothesized that with expanded capacities, public health workers would feel more equipped to contribute to changing systems and processes to improve wellbeing. Across the interviews, respondents articulated how they believe completing PHE benefited them. Beyond feeling more equipped with stronger knowledge and expanded skills, they described shifts in their understanding of public health and their role within it. Respondents described that PHE helped them gain a broader perspective of public health, including a stronger emphasis on prevention, community engagement, collaboration, and being responsive to actual (not presumed) needs. For example: *“[Before PHE] I wasn’t thinking about the other factors that come into play. It is not just whether or not someone has access to the money to purchase these foods. [It is] do they have access to get to the grocery store? Where’s that located? What other things are happening within their household that might be interfering with that or what environmental factors come into play that create barriers and challenges for them? [Before] I was narrowly focused, I think, on my approach.”*

Respondents shared case examples of how they have translated their capacity into actions linked to engagement, network-building, assessment, and program planning, and reflected on how they believe their improved work has resulted in stronger relationships between organizations and community members, improved programs that address community needs (e.g., increased food access, overcoming transportation or access barriers), and growing trust in public health initiatives. For example: “*I wasn’t expecting how much more broadly it helped me to think about the work that I’m doing, the partnerships that I have that I would not have necessarily included in conversations about public health that I should have been thinking about--everything that they do.”* Respondents also shared that they believe these outcomes can serve to promote long-term health-focused behaviors, reduce stigma, and support local economies. For example: *“It is making sure that people are collaborating and for the good of the community because it is for the community. It is not for us, it is for the community.”*

## Discussion

Globally, there is a deficit of public health and health care workers: an estimated 12.9 million will be needed by 2035 (42). In the US alone, an estimated 100,000 new public health workers are needed now (4). Public health workforce needs are further strained in the US as an estimated 84% of government public health workers have no formal public health training (15), and more than 50% report skills gaps and training needs (15). As we look to the future of public health, a skilled and interdisciplinary workforce is essential. Existing research on career pipelines and pathways suggests that, in addition to stronger recruiting pipelines from accredited public health programs (43), and stronger workplace policies to engage and retain workers (44), real-time/in-service capacity building will remain a priority (4, 5, 17, 23). However, in strained funding environments, being able to demonstrate outcomes from investments of time and money in workforce development is critical.

This study sought to explore methods to evaluate whether and how public health workforce capacity-building interventions [such as PHE] may translate into meaningful public health action that helps to create conditions where communities can achieve health. While some existing research documents intervention-related gains in capacity and confidence among public health workers, there is a critical gap in exploring the “so what?”: does expanded capacity lead to changes in behaviors or actions that can support positive health impacts? Prior research showed that individuals who participated in the in-depth capacity enhancing intervention, PHE, demonstrated expanded capacities in 54 core competencies and strategic skills (14). This study took this a step further. Developing a theory of change based on existing public health workforce frameworks allowed us to articulate potential pathways to impact and helped to inform a research design.

Application of qualitative methods helped us explore these pathways and identify themes to explore more broadly. We sampled a sub-set of PHE graduates and invited them to describe how investments in their own *capacity* have impacted their work. Broadly, participants were able to describe translation of knowledge and skills into improved *capabilities*. Coding elucidated a series of individual behaviors and actions that participants apply in their work; these were aligned with the *Core Competencie*s (8) and *Strategic Skills* (28) frameworks, and began to touch on some of the *Essential Services* (29). For example, participants described deep investments in communication and collaboration and applying systems thinking and leadership to inform data access and use and program planning. Coding also elucidated collective or team-based actions that participants apply in their work; these aligned with the *Essential Services* (29). For example, participants described working to strengthen public health systems, to better align programs to community needs, and to ensure trust and confidence in the public health system. Finally, participants also described the impetus for their work, or the *changes in conditions* that they seek. Broadly, these aligned with the goals and mission of public health: to focus on disease prevention, to improve the social drivers of health, and to work toward health equity. These themes are encouraging, given today’s public health focus and priorities, where current literature suggests that cross-sector collaboration is critical to improving public health (3, 45, 46), and that this is supported by the application of core competencies and strategic skills (3, 6, 47–49).

**Figure 2:**
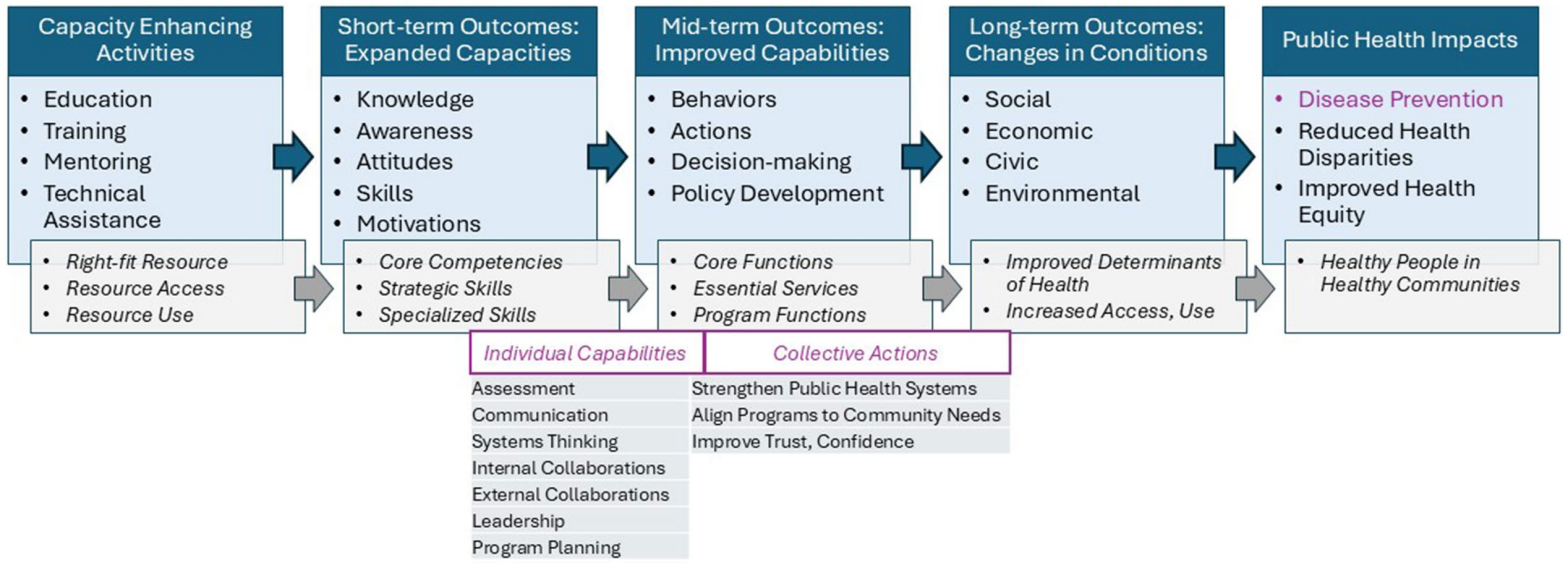
Modified theory of change—translating capacity building into action.

Using a theory of change framework and qualitative methods, we were able to explore how expanded knowledge and skills among practitioners led to improved individual capabilities, collective organizational actions, and changes in community conditions. For our specific case study, we were able to elucidate how learners see and describe changes in their *Individual Capabilities*, and how these contribute to *Collective Actions*. We were able to see how the reported capabilities aligned well with competencies and services expected of the workforce (e.g., Core Competencies for Public Health Professionals and/or Strategic Skills domains such as communication, systems thinking, collaboration, and leadership), and how those led to both individual actions and team-based/collective actions aligned with the Essential Services (e.g., assessment, planning, strengthening systems, building trust; Figure 2). These findings suggest that when interventions are thoughtfully designed and delivered, they can catalyze shifts in practice—enhancing collaboration, communication, leadership, and systems thinking. These capabilities support stronger public health systems, better alignment of programs to community needs, and efforts to rebuild public trust. Importantly, participants linked their work to broader goals such as improving social determinants of health, advancing prevention, and promoting equity. Further this work showed that themes reported by participants in open-ended interviews aligned closely with existing public health frameworks. Although just a pilot, this suggests that existing public health competency statements and frameworks could be used as measures or indicators to help standardize outcome evaluation methods across jurisdictions.

Although presented against the US frameworks of the Strategic Skills, Core Competencies, and Essential Services, the approach used in this study surfaced themes that are consistent with international public health frameworks and could easily be adapted. Further, this approach suggests that the use of qualitative or ‘storytelling’ methods may be valuable in surfacing richness and unanticipated themes. For example, despite the time investment, the rich data we collected allowed us to develop an updated theory of change to guide future evaluation processes at a larger scale. The themes that emerged allowed us to map detail and frameworks to each step in the theory of change, and these frameworks provide possible themes, codes, and indicators for longer-term evaluation processes. Further work by an expanded set of researcher-evaluators will strengthen the framework, the approaches, and most importantly, the evidence.

### Looking forward

This study underscores a critical truth: building capacity within the public health workforce is not just beneficial; it is essential. As public health challenges grow more complex, our ability to respond effectively hinges on equipping practitioners with the right skills, frameworks, and confidence to act. Our findings suggest that when capacity-building interventions are thoughtfully designed and delivered, they can catalyze meaningful changes in individual behavior, organizational practice, and community outcomes. But we must go further. To truly advance the field, we need to keep piloting and refining evaluation methods that capture not just what participants learn, but how they apply it—and what that means for public health impact. Developing scalable, grounded evaluation approaches to assess outcomes will be key to sustaining investment, guiding strategy, and ensuring that workforce development efforts are driving us toward a more equitable and resilient public health system.

As in-depth outcomes evaluation is wanting related to public health workforce development efforts (e.g., in contrast to medical education), we invite scholar-practitioners to consider adoption and adaptation of this *Theory of Change* (Figure 2) to help guide medium and long-term evaluations of public health efforts. In this exploratory use case, we depended on an interview-based protocol and questions to elucidate and validate categories and themes, but as a next step, we anticipate alternative use cases that use these categories and themes to inform larger-scale survey-based evaluation that can more rapidly and thoroughly explore how knowledge and skills (*capacities*) are being applied in the workplace (*capabilities*) and for what purpose (*changes in conditions*), complemented by structured short-answer questions that still invite storytelling. Doing so will support evaluation framed around current public health frameworks (e.g.*, strategic skills*) where skills or performance gaps are known and also help to describe the effects or impacts being seen as a result of expanded capacity. We posit that shared use of this emerging framework for grounded mixed methods evaluation will help develop a collective body of research that shows best practices, value, and impacts of public health capacity-building initiatives. This is especially important given the ubiquity of workforce challenges such as burn-out and erosion of trust. With a shared impact framework, all capacity-building evaluations can start aligning questions related to retention, leadership development, and improved trust.

### Limitations

Limitations of this study include lack of generalizability due to both the small sample size, and to purposive recruitment of select learners who participated in PHE. Code frequencies are only a measure of how often a respondent talked about a theme related to their project and therefore may reflect their biases. However, despite being self-reported, the frequency of theme occurrence across contexts may reveal important trends about how capacity translates into action.

## Conclusion

Evaluation of workforce capacity-enhancing efforts is crucial. This study reinforces what many in the field intuitively know: when we invest in the public health workforce with intention and structure, we see meaningful returns. While it may be too soon to identify measurable impacts in the cases reported herein, the potential for these is clear. Through PHE, participants not only gained knowledge and skills, but they have also translated those gains into real-world actions that align with the *Essential Services* of public health. Their stories reflect a shift in mindset, a deepened understanding of systems and equity, and a commitment to community-centered practice. To sustain and scale these gains, the field must continue piloting and refining evaluation methods that capture not only learning outcomes but also real-world application and impact.

While qualitative methods like those used here are resource-intensive, they offer rich insight into how capacity building can catalyze change. As we look ahead, developing scalable, grounded evaluation strategies will be critical—not only to demonstrate impact, but to ensure that our workforce development efforts are truly advancing the mission of public health: to create conditions in which all people can thrive. There are many different public health capacity building initiatives that are funded, developed, and promoted each year and we encourage a shared evaluation framework across all trainings to ensure proper comparison and documentation of material changes to the workforce as a result of successful participation. The pilot evaluation framework presented in this paper, upon further development and refinement, may prove a useful tool in assessing the actions of public health workers, and may help guide future assessments in identifying key actions requisite for effective public health interventions. Developing grounded, scalable approaches to assess workforce development outcomes is essential for guiding investment, informing strategy, and ensuring that public health capacity-building efforts in both the US and other nations are driving meaningful changes.

## Data Availability

The original contributions presented in the study are included in the article, further inquiries can be directed to the corresponding author.
